# The CCR5-antagonist Maraviroc reverses HIV-1 latency *in vitro* alone or in combination with the PKC-agonist Bryostatin-1

**DOI:** 10.1038/s41598-017-02634-y

**Published:** 2017-05-24

**Authors:** María Rosa López-Huertas, Laura Jiménez-Tormo, Nadia Madrid-Elena, Carolina Gutiérrez, Sara Rodríguez-Mora, Mayte Coiras, José Alcamí, Santiago Moreno

**Affiliations:** 1 Department of Infectious Diseases, Hospital Ramón y Cajal, Alcalá de Henares University, Instituto Ramón y Cajal de Investigación Sanitaria (IRYCIS), Madrid, Spain; 2 AIDS Immunopathology Unit, National Center of Microbiology, Instituto de Salud Carlos III, 28220 Majadahonda, Madrid Spain

## Abstract

A potential strategy to cure HIV-1 infection is to use latency reversing agents (LRAs) to eliminate latent reservoirs established in resting CD4+ T (rCD4+) cells. As no drug has been shown to be completely effective, finding new drugs and combinations are of increasing importance. We studied the effect of Maraviroc (MVC), a CCR5 antagonist that activates NF-κB, on HIV-1 replication from latency. HIV-1-latency models based on CCL19 or IL7 treatment, before HIV-1 infection were used. Latently infected primary rCD4+ or central memory T cells were stimulated with MVC alone or in combination with Bryostatin-1, a PKC agonist known to reverse HIV-1 latency. MVC 5 μM and 0.31 μM were chosen for further studies although other concentrations of MVC also increased HIV-1 replication. MVC was as efficient as Bryostatin-1 in reactivating X4 and R5-tropic HIV-1. However, the combination of MVC and Bryostatin-1 was antagonistic, probably because Bryostatin-1 reduced CCR5 expression levels. Although HIV-1 reactivation had the same tendency in both latency models, statistical significance was only achieved in IL7-treated cells. These data suggest that MVC should be regarded as a new LRA with potency similar as Bryostatin-1. Further studies are required to describe the synergistic effect of MVC with other LRAs.

## Introduction

The major reason why human immunodeficiency virus type 1 (HIV-1) is currently incurable is the persistence of latent reservoirs that cannot be eliminated despite antiretroviral treatment (ART)^1–4^. Latent reservoirs *in vivo* are diverse and consist of several T cell populations including stem central, transitional and effector memory CD4+ T cells^5, 6^. The HIV-1 genome is stably integrated into the host DNA of these cells but viral genes are not expressed at significant levels due to the absence of cellular transcription factors required for the activation of the HIV-1 promoter LTR (long terminal repeat). HIV-1 latent reservoirs are small but extremely stable and long-lived even under different treatment regimens^1, 2, 7^. Although this latent state escapes from ART and is undetectable to the immune system, it can be reversed upon cellular activation allowing the production of replicative competent viruses, the replenishment of the reservoir and viral spread^8–10^. Therefore, a plausible strategy to eliminate the reservoir and achieve the HIV-1 functional cure is the use of pharmacological drugs to activate viral gene expression in latently infected cells. However, to avoid undesirable side effects, only approaches that trigger HIV-1 reactivation with minimal cellular activation or cytokine production may be considered for therapeutic use^11, 12^.

Because of the low frequency of latently infected cells in patients on ART, estimated as being one replication-competent latent provirus per million of resting CD4+ (rCD4+) T cells^13, 14^, the use of *in vitro* models of latency is essential in the screening for new latency reversing agents (LRAs). Models of post-integration latency may allow efficient viral integration while maintaining low levels of viral expression in rCD4+ T cells. As it is unknown which model is most relevant or harbors the best physiological mechanism of latency, different models may be considered as complementary^15^. Commonly used latency models are Jurkat-Lat cells^16^, Bcl-2 transduced primary CD4+ T cells^17^, cultured central memory T (TCM) cells^15^ and CCL19-treated primary rCD4+ T cells^18–20^. Recently, we have described a post-integration latency model based on IL-7 treatment of primary rCD4+ T lymphocytes^21^. Multiple LRAs have been identified using these models. However, a comparative *ex vivo* evaluation of several drugs disrupting latency concluded that none of them could singly disrupt HIV-1 latency, except for a moderate reactivation mediated by the protein kinase C (PKC) agonist Bryostatin-1^22^. As a result, there is a general assumption that the combination of anti-latency compounds is required to effectively and completely induce the replication of all competent proviral genomes of the HIV-1 reservoir *in vivo*. Accordingly, a synergistic effect of bryostatin-1 with histone deacetylase inhibitors (HDACi) or with bromodomain inhibitor JQ1 has been shown both *in vitro* models and in *ex vivo* cells from patients on ART^23–26^.

Maraviroc (MVC) is the only CCR5 antagonist currently approved by the United States Food and Drug Administration, the European Commission, Health Canada, and several other countries for the treatment of patients infected with R5-tropic HIV-1^27^. MVC inhibits the binding of chemokines CCL3, CCL4 and CCL5 to CCR5 resulting in a potent inhibition of CCR5 downstream signaling but without internalization of the receptors^28^. The drug binds to a CCR5 area different from the binding site used by chemokines and HIV-1 gp120 protein. It changes the receptor conformation so that interactions with the virus and the subsequent entry into the host cell are inhibited^29–32^. Apart from its antiviral effect, MVC induces immunological changes beneficial for HIV-1 disease and produces an increase of immune cells such as CD4+ and CD8+ lymphocytes as well as a deficient activation of T cells^33–35^. The majority of patients with R5-tropic viruses are selected during transmission and persists during primary HIV-1 infection when the latent reservoir is established^36^. Consequently, the intensification of ART with MVC during early infection has been suggested as a potential strategy to limit the size of the reservoir. However, MVC intensification did not yield significant reduction of the latent viral reservoir neither in chronic nor in acute HIV-1 patients *in vivo* ^37–42^. Furthermore, MVC intensification resulted in a transient increase of 2LTR DNA circles^37^, a marker of recent infection events, suggesting this drug may increase viral replication through the transcriptional activation of latent HIV-1. In a clinical trial of MVC intensification conducted on HIV-1 infected adults taking suppressive ART, our group found that MVC increased unspliced viral RNA and it correlated with the enhanced expression of NF-κB dependent genes^43^. Later, other groups have confirmed this finding^44^. NF-κB is a major transcription factor that regulates HIV-1 transcription and replication^45^ and a preferential target of anti-latency drugs targeting PKC pathways.

In this article we have explored the role of MVC as a LRA alone or in combination with Bryostatin-1 using two primary CD4+ T cells latency models. Our data show that MVC reactivates *in vitro* X4 and R5-tropic HIV-1 from latency with similar efficacy than other described agents such as Bryostatin-1.

## Results

### CCL19 and IL-7 enhanced proviral integration in rCD4+ T cells, being IL-7 more efficient

The small size of the latent HIV-1 reservoir limits reactivation studies *in vivo* and highlights the importance of using *in vitro* latency models. In this study, two different latency models based on CCL19 or IL-7 treatment before HIV-1 infection were used. Both latency models have already been described and fulfill the criterion of significantly increasing the proviral integration without outstanding viral replication. The CCL19-based latency model is commonly used^46–48^ while the IL-7-based latency model has recently been described^21^. Its value as a model for viral reactivation has yet to be confirmed.

**Figure 1:**
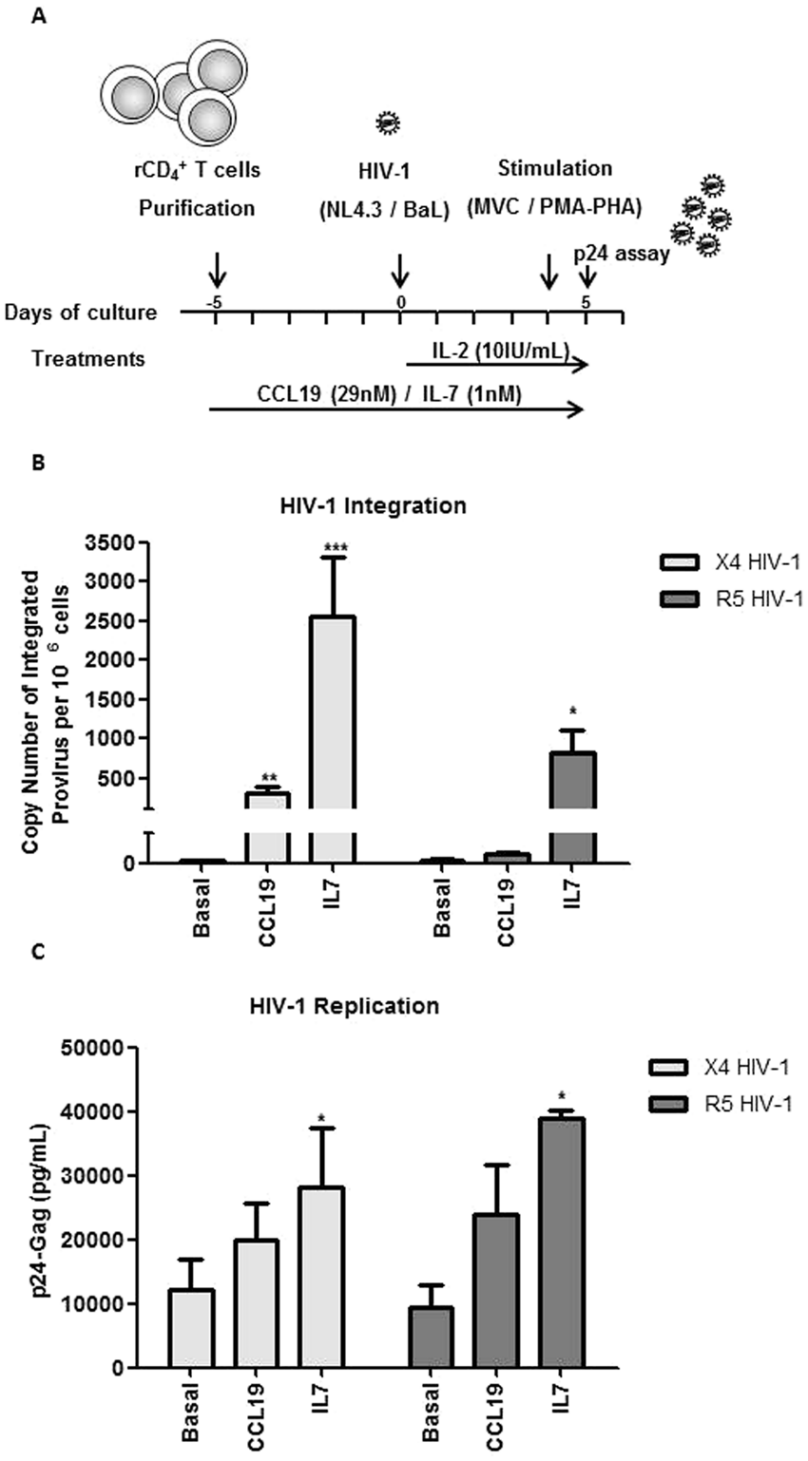
Proviral Integration in HIV-Infected rCD4+ lymphocytes treated with CCL19 or IL-7. CD4+ lymphocytes were treated with CCL19 or IL-7 for 5 days and then infected with X4-tropic NL4–3 strain or with R5-tropic BaL strain and cultured for 5 additional days. (**A**) Scheme of HIV-1 primary cells latency models. (**B**) qPCR analysis of HIV-1 proviral integration. (**C**) HIV-1 basal replication was assessed by quantifying p24/Gag levels in the culture supernatant. Statistical significance was calculated by using Kruskal-Wallis test with Dunn’s Multiple Comparison Test (*p < 0.05, **p < 0.01 and ***p < 0.001). Data are represented as mean ± SEM.

Figure 1A shows a diagram of the latency models used. rCD4+ T cells were treated with CCL19 (29 nM) or IL-7 (1 nM) for 5 days and then infected with X4-tropic NL4.3-wt or X5-tropic BaL strains for an additional 5 days all the while in the presence of CCL19 or IL-7 together with IL-2 (10 IU/mL). Four days after infection, viral integration and basal replication was assessed. Infected cells were then challenged for HIV-1 reactivation using MVC, PMA/PHA as a positive control or left untreated as a negative control.

After 4 days of infection with X4-tropic NL4.3-wt (*p* < 0.01 and *p* < 0.001) (Fig. 1B), the efficiency of proviral integration increased 60- and more than 500-fold in CCL19 and IL-7-treated cells, respectively. Viral integration of R5-BaL viruses was enhanced 3-fold after treatment with CCL19 whereas it increased more than 180-fold (*p* < 0.05) in IL-7 treated cells (Fig. 1B). In contrast with the high integration levels obtained, viral replication was only increased by 2–3 fold as compared to untreated CD4+ lymphocytes in IL-7-treated (*p* < 0.05) (Fig. 1C).

### MVC increases LTR-dependent activation and HIV-1 replication in rCD4+ T cells

**Figure 2:**
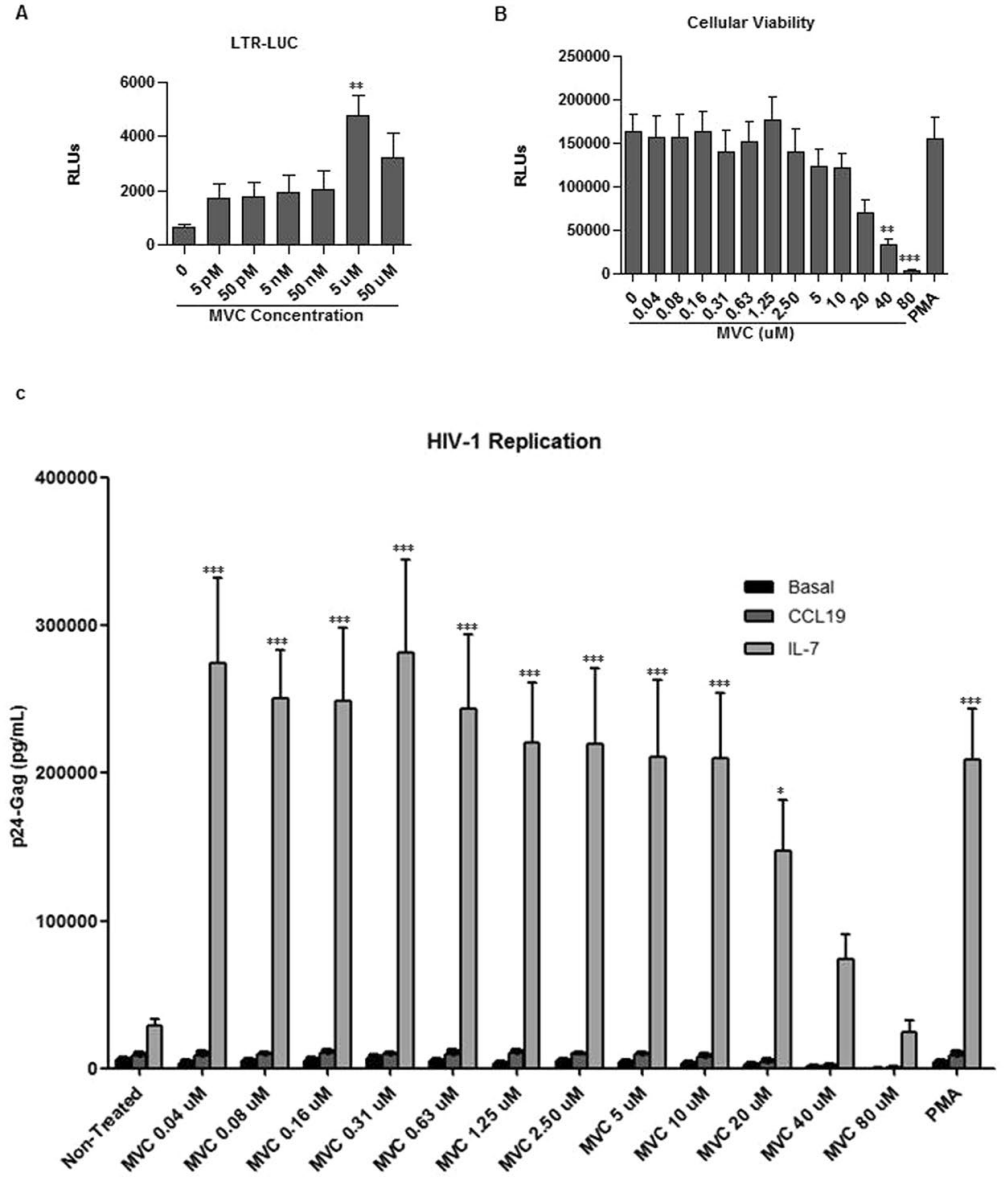
LTR activation and HIV-1 replication in MVC-treated CD4+ T cells. (**A**) Analysis of luciferase expression (RLUs) in CD4+ T cells electroporated with pLTR-LUC vector. MVC was added 4 hr post-transfection and cells were incubated for subsequent 18 hr. (**B**) Cellular viability was measured by chemiluminescence (RLUs) in CD4+ T cells treated with MVC or PMA/PHA for 18 hr. (**C**) Primary CD4+ T cells were treated with CCL19 or IL-7 for 5 days, then infected with X4-tropic NL4–3 strain and cultured for 5 additional days. MVC at indicated concentrations or PMA/PHA were added during the last 18 hr. HIV-1 replication was assessed by quantifying p24/Gag levels in the culture supernatant. Statistical significance was calculated using Kruskal-Wallis test with Dunn’s Multiple Comparison Test (*p < 0.05 and ***p < 0.001) for (**A**) and (**B**) or using two way ANOVA with Bonferroni post-test analysis (**p < 0.01 and ***p < 0.001) for (**C**). All data are represented as mean ± SEM.

Our group and others have described that MVC triggers NF-κB activation and the expression of -κB dependent genes in treated patients^43, 44^. To assess the effect of MVC on HIV-1 transcription rCD4+ T cells were electroporated with a luciferase expression vector under the control of the HIV-1-LTR (pLTR-LUC). Four hours after transfection, cells were incubated with different concentrations of MVC ranging from 25 pM to 50 μM for 18 hours. This wide range of concentrations was chosen considering the *in vitro* inhibitory concentration (IC) IC90 of MVC to inhibit CCR5-tropic viral entry and MVC plasma concentrations of treated patients. IC90 of MVC to block viral entry ranges from 0.5 nM to 13.4 nM, depending on the viral strain used^29^. However, MVC mean plasma concentrations are as high as 700 ng/mL (1.36 μM) and 1100 ng/uL (2.14 μM), in typical treatments of 600 mg once a day or 300 mg twice a day^49^. Among the concentrations tested, LTR-dependent transcription was enhanced 7-fold only in CD4+ T cells treated with MVC 5 μM (p < 0.005) (Fig. 2A).

The correlation between the potency of induction of HIV-1 LTR expression in *in vitro* models systems and the ability of chemical agents to induce viral expression from rCD4+ T cells is not yet completely defined^50^. The effect of different concentrations of MVC in the reactivation of latent NL4.3-wt HIV-1 strain was studied in CD4+ primary T lymphocytes treated with CCL19 or IL-7 before HIV-1 infection. Because MVC 5uM was the most effective concentration at enhancing LTR dependent transcription, two-fold serial dilutions around this value were chosen. MVC ranged from 0.04 μM to 80 μM. MVC treatment was carried out during 18 h and then HIV-1 replication was measured. Treatment with PMA/PHA for 18 h was used as positive control. Cellular viability was monitored in non-infected cells subjected to the same experimental conditions. MVC stimulation dramatically reduced cellular viability when used at 40 μM or more (p < 0.005) (Fig. 2B). HIV-1 replication was slightly enhanced in cells treated with CCL19 but no statistically significant changes were observed (Fig. 2C). IL-7 data could be subtracted from the statistical analysis and then compared with viral replication in CCL19-treated cells and untreated cells. Under these circumstances, MVC 0.16 uM and 1.25 uM enhanced viral replication significantly (p < 0.05 and p < 0.001, respectively). HIV-1 replication from latency established by IL-7 exposure was enhanced 7.4-fold and even more at MVC concentrations ranging from 0.04 uM to 10 μM (p < 0.001) and 5.1-fold at 20 uM (p < 0.05) (Fig. 2C). A higher concentration of MVC (40 μM and 80 μM) enhanced viral replication, but without statistical significance probably because of cellular viability was reduced under these conditions (Fig. 2C). The highest increase in replication was achieved at MVC 0.31 uM (9.8-fold, p < 0.001) (Fig. 2C). PMA/PHA treatment enhanced 7.3-fold HIV-1 replication in CD4+ T lymphocytes latently infected after IL-7 exposure (p < 0.001) (Fig. 2C).

Stimulation with MVC 5 μM was chosen for further studies based on the fact that close plasma concentrations were achieved during patients’ treatments and it was able to significantly induce LTR-dependent transcription together with HIV-1 replication without compromising cellular viability.

### MVC enhances HIV-1 replication with potency similar to Bryostatin-1 in rCD4+ T cells

The combination of LRAs has been suggested as one of the most plausible strategies to induce the complete expression of replication-competent proviral genomes^50^. The effect of MVC in HIV-1 reactivation was studied in combination with different amounts of Bryostatin-1, a PKC agonist that is known to be a LRA. Stimuli were incubated during 18 h before assessing HIV-1 replication.

**Figure 3:**
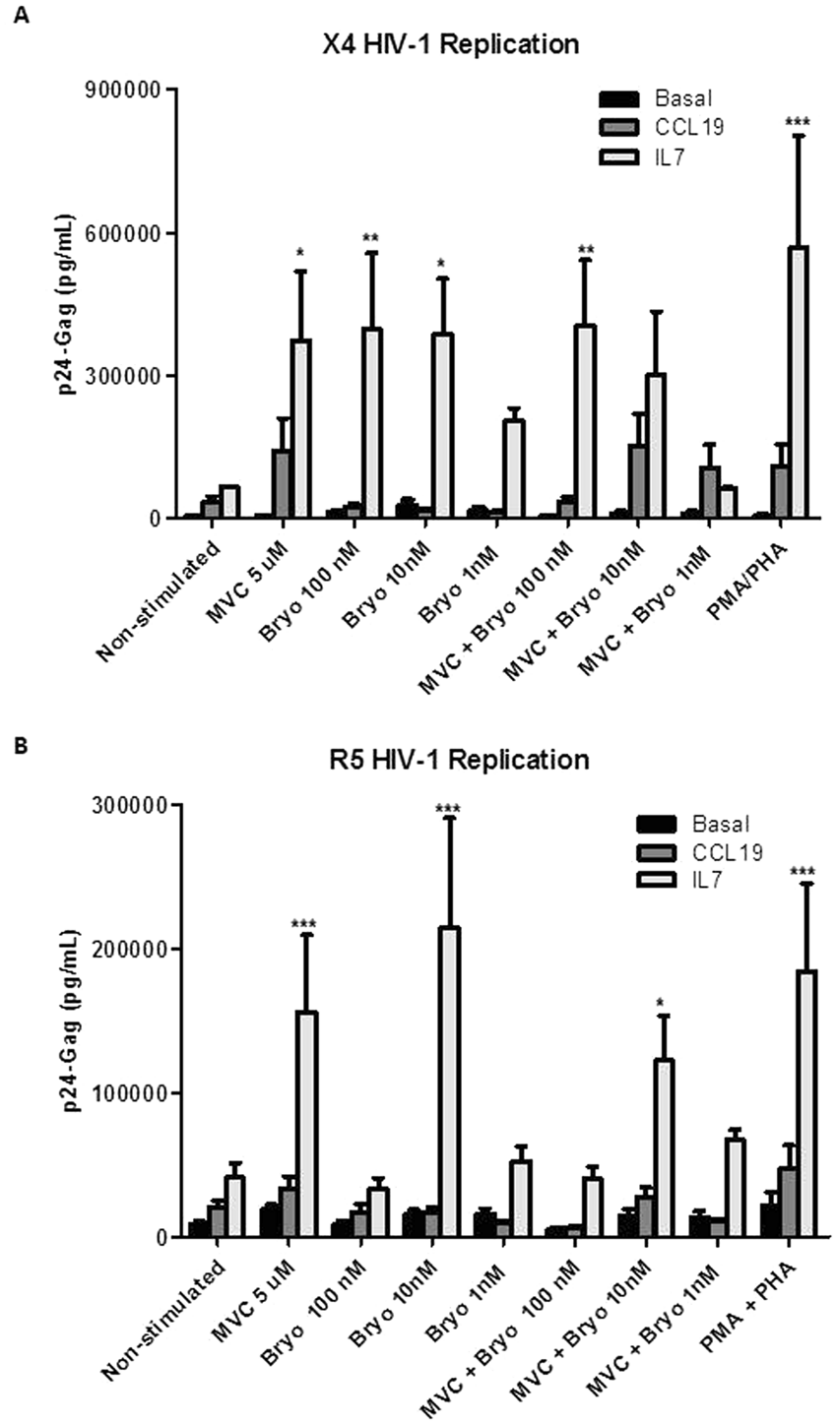
HIV-1 replication in rCD4+ T cells treated with MVC alone or in combination with Bryostatin-1. Primary rCD4+ T cells were treated with CCL19 or IL-7 for 5 days, then infected with X4-tropic NL4–3 strain (**A**) or with R5-tropic BaL strain (**B**) and cultured for 5 additional days. MVC (5 μM) or Bryostatin-1 at indicated concentrations were added alone or in combination during the last 18hr. Stimulation with PMA/PHA was used as positive control. HIV-1 replication was assessed by quantifying p24/Gag levels in the culture supernatant. Statistical significance was calculated using two way ANOVA with Bonferroni post-test analysis (*p < 0.05, **p < 0.01 and ***p < 0.001). Data shown are mean ± SEM.

Bryostatin-1 concentrations as high as 100 nM were previously shown to be non-toxic in T cells *in vitro* ^51^. Bryostatin-1 enhanced around 6-fold the replication of competent X4-tropic HIV-1 virus (NL4.3) in IL-7-treated rCD4+ T lymphocytes when used at 100 nM or 10 nM (6–1 and 5.9-fold, respectively) (p < 0.5, p < 0.01 and p < 0.05, respectively) (Fig. 3A). A similar increase was obtained after MVC stimulation (5.3-fold) (p < 0.05) in IL-7 treated cells (Fig. 3A), suggesting that MVC is a LRA as potent as Bryostatin-1 in this latency model. Furthermore, both compounds were almost as efficient as PMA/PHA stimulation used as a positive control in recovering HIV-1 replication (8.7-fold in IL-7-treated cells). Despite the fact that stimulation with MVC alone or combined with Bryostatin-1 10 nM increased HIV-1 replication in CCL-19 treated-cells, it did not reach statistical significance. A combined treatment of MVC and Bryostatin-1 (100 nM) induced HIV-1 replication in IL-7 treated cells (p < 0.01) (Fig. 3A). Nonetheless, this increase was similar to the effect observed in rCD4+ T cells stimulated alone with MVC or Bryostatin-1 and therefore a synergetic action of both compounds was not found. The combination of MVC with Bryostatin-1 at 10 nM also recovered HIV-1 replication from latency in the IL-7 model. However, this recovery was less efficient than after the administration of MVC alone (Fig. 3A), suggesting a potential antagonism when MVC was combined with low Bryostatin-1 concentrations (Fig. 3A).

The recovery from latency of R5-tropic HIV-1 viruses had several similarities but also discrepancies. MVC reversed HIV-1 latency 3.8-fold in IL-7 treated cells (p < 0.005) (Fig. 3B). It was less potent than Bryostatin-1 (10 nM), which induced 5.3-fold of HIV-1 replication in IL-7 treated cells (p < 0.005) (Fig. 3B). However, Bryostatin-1 100 nM was inefficient to induce viral replication. The combined treatment of MVC and Bryostatin-1 (10 nM) also enhanced viral replication in rCD4+ T cells from the IL7 latency model but in less quantity than individual treatments (p < 0.05) (Fig. 3B), further suggesting the non-synergistic but antagonistic effect of the combined treatment.

**Figure 4:**
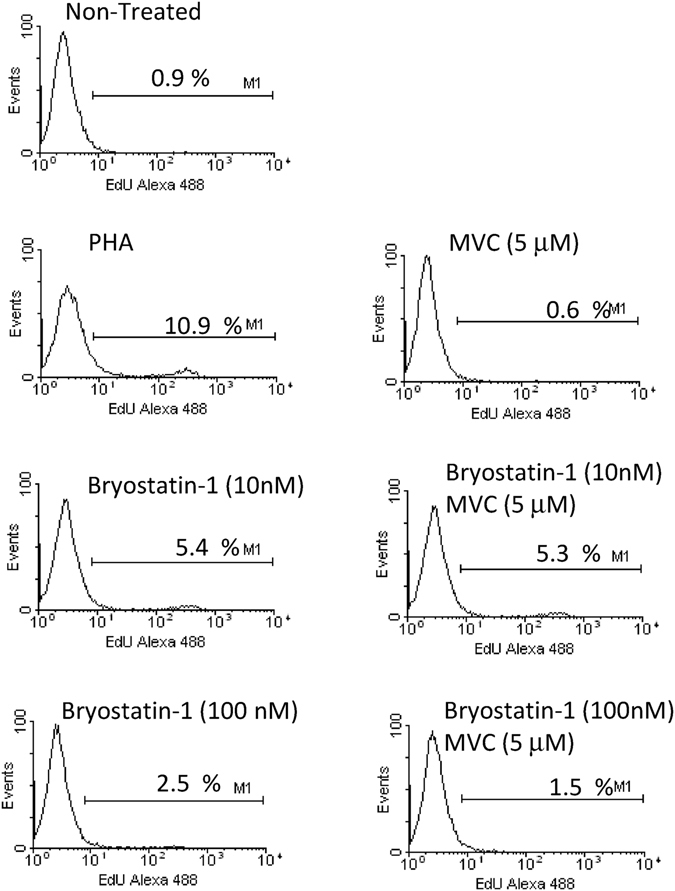
Analysis of cell proliferation in rCD4+ T treated with MVC alone or in combination with Bryostatin-1. Primary rCD4+ T cells were left untreated or treated with MVC (5 μM) alone or in combination with Bryostatin-1 (10 nM or 100 nM) for 18 h. Non-treated cells were used as a negative control. Stimulation with PMA for 18 h was used as positive control. Cell proliferation was measured by quantifying the percentage of DNA-synthesizing cells. Data shown are cytometry histogram of FL-1 channel fluorescence. Numbers indicate the percentage of proliferating cells. A representative experiment is shown.

The cellular ability to proliferate was studied under these experimental conditions (Fig. 4). PHA stimulation during 18 h was used as a positive control. The percentage of proliferating rCD4 cells was not altered after incubating with MVC (5 μM) for 18 h but slightly increased up to 5.4% and 2.5% after Bryostatin-1 10 nM and 100 nM treatment, respectively. This increase remained almost the same when MVC and Bryostatin-1 were added together.

### MVC reverses X4 and R5-HIV-1 latency in TCM cells

**Figure 5:**
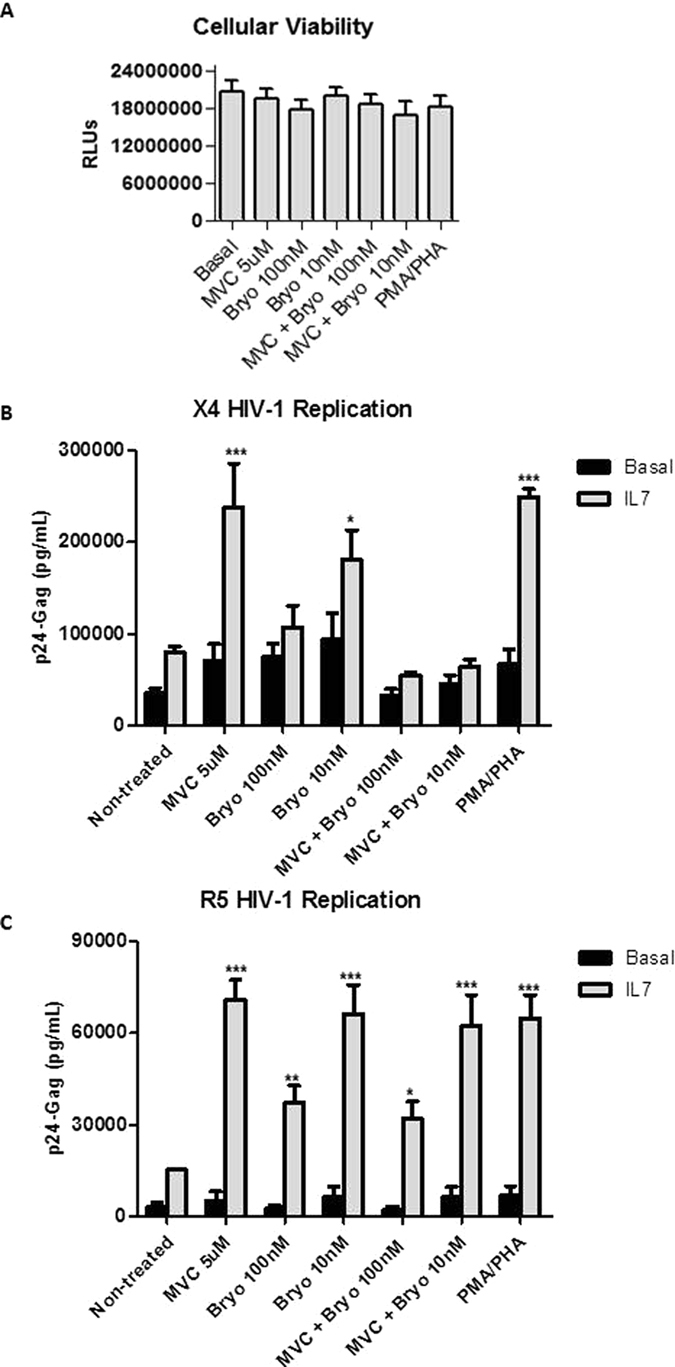
HIV-1 replication in TCM cells treated with MVC alone or in combination with Bryostatin-1. Primary TCM cells were treated with CCL19 or IL-7 for 5 days and then infected with X4-tropic NL4–3 strain or with R5-tropic BaL strain and cultured for 5 additional days. MVC (5 μM) alone or in combination with Bryostatin-1 (100 nM or 10 nM) was added during the last 18 hr. Stimulation with PMA/PHA was used as positive control. (**A**) Cellular viability was measured. Cell viability was measured by chemiluminescence (RLUs) in CD4+ T cells treated with MVC, Bryostatin-1 or PMA/PHA during 18 hr. (**B**) X4 HIV-1 and (**C**) R5 HIV-1 replication was assessed by quantifying p24/Gag levels in the culture supernatant. Statistical significance was calculated using Kruskal-Wallis test with Dunn’s Multiple Comparison Test for (**A**) and two-way ANOVA with Bonferroni post-test analysis for (**B**) and (**C**) (*p < 0.05 and ***p < 0.001).

TCM cells have been identified as the main cellular HIV-1 reservoir *in vivo* ^6^. TCM cells were purified from healthy donors and subjected to IL-7-based *in vitro* latent infection with HIV-1 strains NL4.3-wt and BaL. HIV-1 strains NL4.3-wt and BaL with respectively X4 and R5-tropic viruses were used. Before HIV-1 infection, cellular viability of TCM cells treated with MVC (5 μM) or Byrostatin-1 (10 nM, 100 nM) or a combination of both was analyzed and non-significant changes were found after 18 hours of stimulation (Fig. 5A). MVC reactivated 3.0 and 4.3-fold HIV-1 replication of competent X4 and R5 viruses, respectively, in TCM (p < 0.001) (Fig. 5B and C). Similar recuperation of viral replication was achieved in a positive control of TCM cells treated with PMA/PHA. Bryostatin-1 stimulation was less efficient than MVC in reactivating the NL4.3 wt strain and only 10 nM concentration was significant (2.3-fold, p < 0.05) (Fig. 5B). The combination of MVC with Bryostatin-1 was unable to induce X4 HIV-1 replication and had a strong inhibitory effect on viral replication (0.7-fold decrease) (Fig. 5B). Bryostatin-1 led a different profile of viral reactivation in latently infected TMC cells with the R5-tropic HIV-1 BaL strain. Bryostatin-1 was as efficient as MVC or PMA/PHA when used at 10 nM inducing a 4.4-fold increase in viral reactivation (p < 0.001) (Fig. 5C). However, Bryostatin-1 100 nM enhanced viral replication only 2.4-fold (p < 0.05) (Fig. 5C). Similar HIV-1 reactivation of 2.1- and 4.1-fold increase was achieved when using a combination of MVC and Bryostatin-1 100 nM or 10 nM, respectively (p < 0.05 and p < 0.001) (Fig. 5C). Therefore, neither a synergistic nor an antagonistic effect of the combined treatment was shown in R5 HIV-1 reactivation.

### Lower doses of MVC did not synergize with Bryostatin-1 to activate HIV-1 replication in rCD4+ T cells

A wide range of MVC concentrations that were lower than 5 μM enhanced viral replication with similar efficiency (Fig. 2). Because Bryostatin-1 only synergizes with other LRAs to induce HIV-1 transcription when used at 1 nM concentration but not at higher doses^24^, we wondered if the same situation may account for MVC.

**Figure 6:**
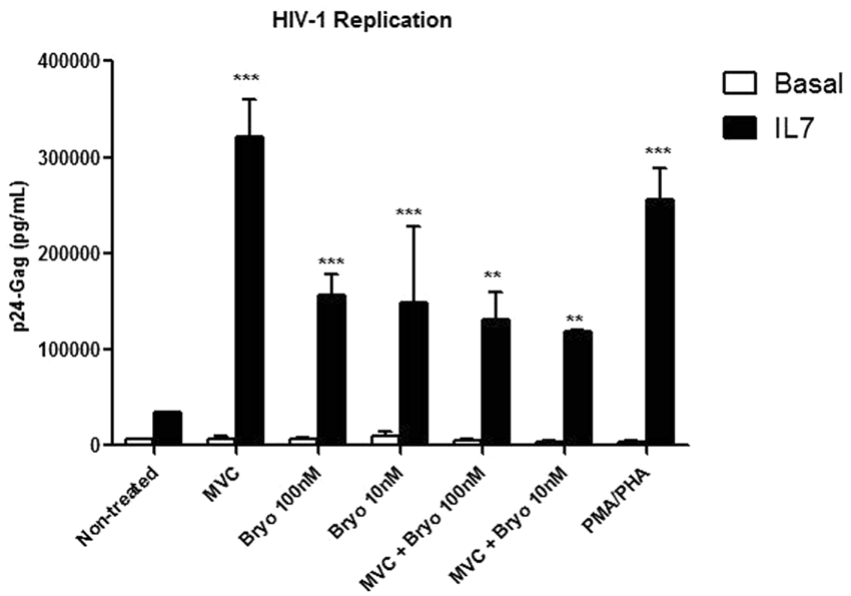
HIV-1 replication in rCD4 cells treated with MVC 0.30 μM alone or in combination with Bryostatin-1. rCD4+ T lymphocytes were treated with IL-7 for 5 days and then infected with X4-tropic NL4–3 strain and cultured for 5 additional days. MVC (0.31 μM) alone or in combination with Bryostatin-1 (100 nM or 10 nM) was added during the last 18 hr. Stimulation with PMA/PHA was used as positive control. HIV-1 replication was assessed by quantifying p24/Gag levels in the culture supernatant. Statistical significance was calculated using two-way ANOVA with Bonferroni post-test analysis (**p < 0.01 and ***p < 0.005).

MVC 0.31 μM, which is more than 16-fold lower than 5 μM, was the concentration chosen for this study. The effect of MVC 0.31 μM in X4-tropic HIV-1 reactivation was studied in combination with Bryostatin-1 10 nM or 100 nM in latently infected rCD4 T lymphocytes. The IL-7 based model was used. Stimuli were incubated during 18 h before assessing HIV-1 replication. In IL-7 treated cells, viral replication was enhanced 9.4-fold after MVC (0.31 μM) stimulation (p < 0.001) and 4.6-fold after stimulation with Bryostatin-1 (100 nM) (p < 0.001) (Fig. 6). The combined treatment of MVC (0.31 μM) and Bryostatin-1 did not synergize but antagonized viral replication. Reactivation values were similar to those achieved after only stimulating with Bryostatin-1 100 nM (3.8-fold, p < 0.001) (Fig. 6). Similar results were obtained when Bryostatin-1 was used at 10 nM (p < 0.005) (Fig. 6). Therefore, the combined effect of MVC and Bryostatin-1 was antagonistic in rDC4 T cells regardless of MVC concentration.

### MVC and Bryostatin-1 combined treatment reduces CCR5 expression in rCD4+ T cells

In an attempt to find the molecular mechanism responsible for the antagonistic action of the combined treatment of MVC and Bryostatin-1 on HIV-1 replication, CCR5 expression levels were studied in basal and IL7-treated rCD4+ T lymphocytes. Comparisons were performed between untreated cells and cells treated with LRAs.

**Figure 7:**
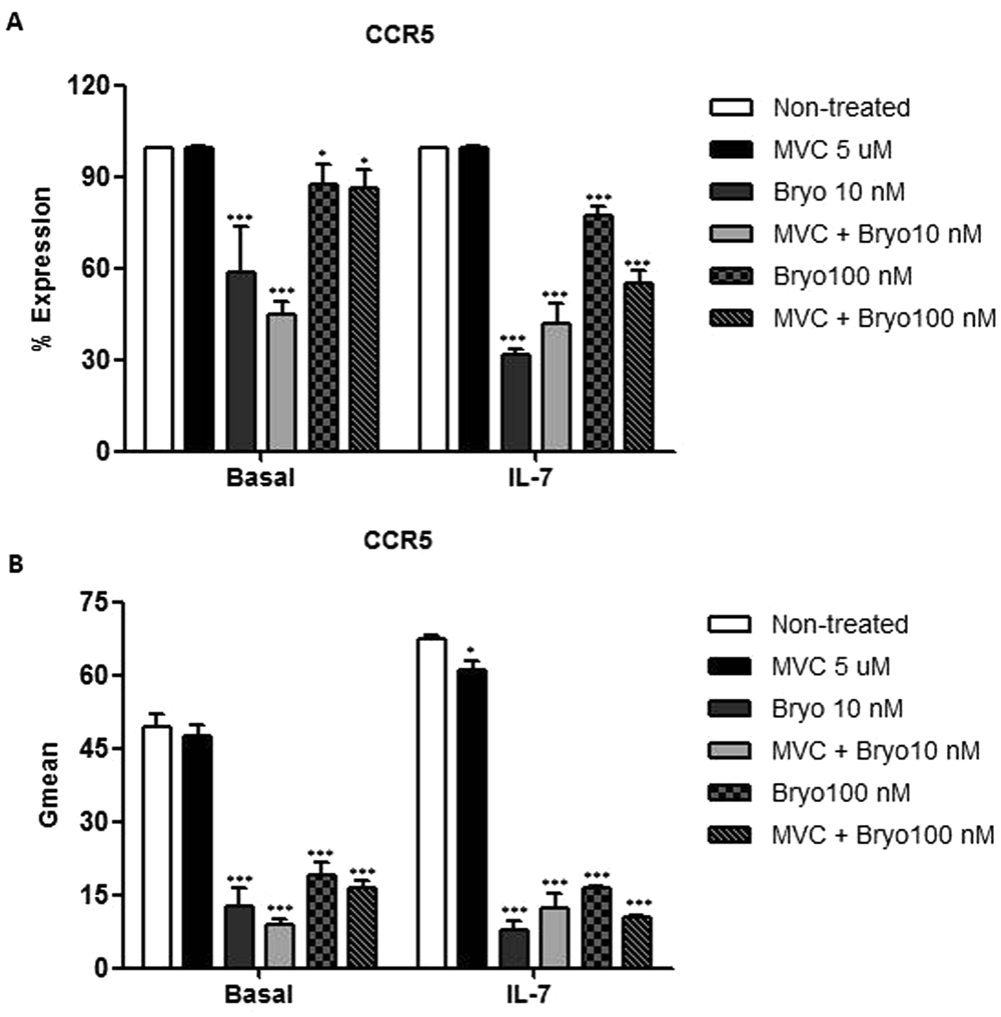
CCR5 expression levels on rCD4 cells treated with MVC or Bryostatin-1. rCD4 cells were treated with IL-7 for 10 days and then stained with a monoclonal antibody against CCR5 conjugated with PE. Non-treated cells were used as referred control. Signals corresponding to PE were analyzed by flow cytometry in FL2 channel. (**A**) The percentage of cells expressing CCR5 on the cell surface and (**B**) the geometric mean fluorescence intensity are represented after subtracting the isotope control. Statistical significance was calculated using two way ANOVA with Bonferroni post-test analysis (*p < 0.05, **p < 0.01 and and ***p < 0.001). Data shown are mean ± SEM from four independent experiments.

MVC (5 μM) did not change CCR5 expression percentage neither in basal nor in IL-7 treated cells (Fig. 7A), in agreement with previous cytometry data showing that MVC failed to induce CCR5 internalization^29^. Bryostatin-1 (10 nM) reduced in 1.7-fold the expression of CCR5 on the surface of untreated rCD4 cells (Fig. 7A, left) (p < 0.001). CCR5 levels remained 2.2-fold reduced, when MVC (5 μM) was added together with Bryostatin-1 (10 nM) (Fig. 7A, left) (p < 0.001). A similar situation was found when using Bryostatin-1 at 100 nM but differences were less significant (p < 0.05). The effect of Bryostatin-1 on reducing CCR5 expression was more profound in IL-7 treated cells (Fig. 7A, right). Bryostatin-1 used at 10 nM or 100 nM reduced CCR5 expression 3.1-fold and 1.3-fold, respectively and 2.4-fold and 1.8-fold, respectively when administrated in combination with MVC at 5 μM (p < 0.001 in all experimental conditions) (Fig. 7A, right).

The geometric mean (G-mean) fluorescence intensity of cells stained with CCR5 conjugated to PE was measured in the same samples. G-mean was slightly increased after MVC (5 uM) exposure in both basal and IL-7 treated cells (Fig. 7B). This increase was not statistically significant. Both doses of Bryostatin-1 treatment decreased the G-mean of red fluorescence in basal cells (3.9-fold and 2.6-fold reduction respectively for 10 nM and 100 nM) (p < 0.001) (Fig. 7B, left). These G-mean values were enhanced when rCD4 cells were treated also with MVC (5 μM) but were still 5.6-fold and 3.0-fold lower than values of untreated cells, respectively for 10 nM and 100 nM (p < 0.001) (Fig. 7B, left). A similar response was found in IL-7 treated rCD4 cells. Bryostatin-1 reduced G-mean fluorescence of CCR5 8.8-fold and 4.1-fold, respectively for 10 nM and 100 nM concentrations (p < 0.001) (Fig. 7B, right). Combined treatment of MVC and Bryostatin-1 remained CCR5 G-mean values significantly lower than values of non-treated cells. This reduction was of 5.6-fold and 6.5-fold respectively for Bryostatin-1 10 nM and 100 nM (Fig. 7B, right) (p < 0.001). These results showed that during the combined treatment, Bryostatina-1 dramatically reduced CCR5 levels, lowering the number of receptors available for MVC functions.

## Discussion

The existence of latent HIV-1 infected cells is currently the major barrier for viral eradication. A potential approach to cure HIV-1 infection is to use LRAs to eliminate these latent reservoirs but so far no LRA has been shown to be completely effective and therefore finding new LRAs is of increasing importance. Our findings show that MVC, a CCR5 allosteric inhibitor currently used for R5-HIV-1 strains treatment, should be regarded as a drug for reversing HIV-1 latency.

HIV-1 latency models should fulfill at least two criteria that are the induction of high levels of viral integration and low viral replication^18^. Different post-latency models may be considered complementary as diverse signaling pathways and sites of integration are exploited^15^. Viral reactivation mediated by MVC was studied in two different resting primary T cell models based on CCL19 or IL-7 exposure before HIV-1 infection. Both models have been previously described^18–21^. In the CCL19 model two minor modifications were included so that protocols for cytokines exposure and viral reactivation could be used in both models. First, cells were incubated with CCL19 before HIV-1 infection during 5 days instead of 3 days. It did not affect the dynamics of the model as it exceeded the minimum recommended incubation time. It could not be toxic or detrimental for viral integration as the maintenance of CCL19 in the culture medium during the entire experiment is mandatory to preserve HIV-1 latency^19^. Second, the short lifespan step, based on feeding the cell culture with activated T cells before viral reactivation, was suppressed. In addition, the short lifespan in CCL19-treated cells would have increased viral replication but experiments would have also been more time-consuming. Although HIV-1 replication showed the same tendency in both models statistical significance was only reached in IL-7-treated lymphocytes. This could be the result of efficient integration but further studies are required to confirm this hypothesis. Furthermore, IL-7 induces the phosphorylation of the restriction factor SAMHD1, abrogating its antiviral activity, increasing viral reverse transcription and therefore rending CD4+ T lymphocytes more susceptible to the establishment of latent HIV-1 infection events^21^.

The role of IL-7 in regulating HIV-1 latency and reactivation is controversial. IL-7 was firstly proposed as an anti-latency agent due to its ability to increase HIV-1 replication from latency in different contexts, including the reactivation of viral production from latent HIV-1 cellular reservoirs of infected individuals on antiviral therapy^52–54^. However, later studies showed that administration of IL-7 failed to induce HIV-1 replication in latently cells, both *in vitro* and *in vivo* during clinical trials in HIV-1 infected patients; and it also induced higher HIV-1 DNA copies together with clonal expansion of CD4 + lymphocytes^55–58^. Accordingly, it was concluded that IL-7 treatment contributed to maintain the size of HIV reservoirs both *in vitro* and *in vivo* ^6, 57^. Therefore, the stability of the HIV-1 reservoir size has been associated with increased IL-7 concentrations in plasma^6^. A plausible explanation to understand how IL-7 maintains the size of the reservoir is that IL-7 induces only a slight increase of NF-κB activity^21, 57^, insufficient to reverse viral latency in CD4+ lymphocytes but enough to mediate homeostatic proliferation^57^. Moreover, the suboptimal activation of IL-7 may account for the fact that activated HIV-1 infected cells returned to a quiescent status after incubation with low doses of IL-7^59^, similar to those used in this article. The IL-7 HIV-1 latency model was suggested in 2016 on the bases that IL-7 plays three essential roles in HIV-1 persistence^21^: first, reservoir seeding through SAMHD1 phosphorylation; second, reservoir maintenance through homeostatic proliferation; and finally, low ongoing viral replication through incomplete NF-κB activation. The study described for the first time that the inactivation of SAMHD1 restriction factor is the molecular mechanism underlying IL-7-mediated reservoir integration^21^. Additional mechanisms that remain to be elucidated should also be involved. In our hands both models were equally reproducible and had a similar cost. However, IL-7 model shows the advantage of increasing proviral integration at a higher amount probably due to the inactivation of SAMHD1 restriction factor that would result in increased reverse transcription and enhanced integration^21^. It remains to be described if integration site also favours viral replication. Besides, IL-7 treatment does not require a short lifespan to recover HIV-1 production and thus it shortens the protocol performance as discussed above.

Using theses latency models, our data show that MVC is an inducer of HIV-1 replication with either X4 or R5-tropic viruses in rCD4+ and TCM cells, the latter a major cellular reservoir for HIV-1 *in vivo* ^6^. Because a wide range of MVC concentrations were effective to enhance viral replication in IL-7-treated cells, understanding MVC pharmacodynamics is important for future *in vivo* interventions. MVC 5 μM was chosen for further studies on the bases that it could increase LTR activation without cellular toxicity. *In vitro*, MVC is active at low nanomolar concentrations to block the binding of cognate β-chemokine, as MIP1α, MIP1β and Rantes to CCR5^29^. Similar concentrations are required to inhibit the entry of HIV-1 R5-tropic strains^29^. However, the typical dosage of MVC is 300 mg/kg twice daily27 results in a maximum plasma concentration of 1100 ng/μL (2.14 μM), which is achieved two hours post-treatment^49^. The MVC 5 μM used in our study was a higher concentration. However, 5 μM MVC is susceptible to be reached in *in vivo* assays as total MVC exposure at steady-state of 7 days is reported as 4,497 and 5,341 ng · h/mL^60, 61^. At these plasma concentrations, MVC was well tolerated and no safety concerns were observed. Altogether these data suggest that the use of MVC drug regimens currently prescribed could also be clinically plausible for latency reversal.

Independently of its antiviral action on R5 variants, MVC displays immune functions that may be relevant for HIV-1 disease progression and treatment, including an impaired immune activation through the reduction of activation markers as CD25 and HLA-DR^34, 37^. However, MVC-treated HIV-1 patients showed an enhanced expression of −kB target genes including CD69^34, 37, 44^. MVC mediates the activation of NF-κB transcription factor through the direct interaction with its receptor CCR5^44^, supported by the fact that the drug has no significant affinity for other receptors^62, 63^. Due to NF-κB activation, gene transcription depended on the activation of the LTR HIV-1 promoter was enhanced after MVC exposure and it resulted in improved viral replication. This finding could explain that clinical trials of ART intensification with MVC failed to reduce the reservoir size as only a non-significant decay in the total size of latently infected CD4+ T lymphocytes was found *in vivo* ^38–41^. MVC concentration of 5 μM used in most of the experiments of this study resulted in no toxicity *in vitro* and was under the concentration required for mediating cell proliferation^28^. Furthermore, the transcriptomic profile of CD4+ T lymphocytes from MVC-treated patients remained unchanged, except for a non-significant tendency to reduction of TNF gene expression^64^, which was later confirmed through *in vitro* studies^65^. This suggests that cytokine release remains stable after MVC exposure. Based on data showing that MVC increases HIV-1 replication without resulting in T cell activation or cellular toxicity, we suggest that it could be a new LRA and should be considered for therapeutic actions most likely in combination with other agents. However, *ex vivo* assays which use primary rCD4+ T cells recovered directly from HIV-1 infected individuals should be assessed as the next step.

As single LRAs are relatively inefficient at reversing latency *ex vivo* ^22, 66, 67^, it is currently accepted that the combination therapy including diverse reversal drugs, which exploit different cellular pathways, is needed to efficiently reverse latency. Combinations with the PKC agonist Bryostatin-1 has been proposed. Combination of Bryostatin-1 with JQ1 or with HDACi potentiates HIV-1 reactivation *in vitro* and *ex vivo* through a synergistic action that is higher than the addition of independent effects and comparable to the maximal chemical activation^23, 25, 26^. Recently, a double-blind phase I clinical-trial has shown the safety of Bryostatin-1 at single doses of 10 or 20 μg/m in HIV-1 infected patients currently under ART^68^. We have evaluated the action of a combined treatment of MVC with different doses of Bryostatin-1 on HIV-1 reactivation and also compared the potency of both drugs when acting alone *in vitro*. The interactions of MVC with ART and non-HIV-1 drugs have been extensively studied and cannot be predicted^69^. We determined that MVC was a potent inducer of viral replication and achieved potency similar to Bryostatin-1. However, the combination of MVC and Bryostatin-1 was not synergistic at all despite that both pathways merged at NF-κB activation^43, 44, 70^. The interaction was physiological antagonism and it was shown more clearly during X4-tropic viral replication, despite the preserved cell viability. Bryostatin-1 slightly increased cell proliferation, in agreement with previous reports^71^. However, changes in viral replication found in rCD4 T cells treated with both MVC and Bryostatin-1 were not due to alterations of cell proliferation as it remained the same as with Bryostatin-1 alone. Apart from its viral reactivating effects, Bryostatin-1 also exerts an antiviral activity through decreasing the levels of HIV-1 receptors CD4 and CXCR4 at the cell surface^23, 51, 72^. Few data are available describing the effect of Bryostatin-1 on CCR5 expression and only a very slight and non-statistically significant increase was described in CD4 cells^51^. Our results disclosed a dramatic decrease of CCR5 expression levels after Bryostatin-1 stimulation that cannot be reversed when MVC is co-administrated. Consequently, the number of CCR5 receptors available for MVC actions is reduced during the combined treatment with Bryostatin-1 and it may partially account for the antagonism found. However, further studies are needed for a better understanding of associations between these drugs.

Our results show the activity of MVC as an HIV-1 latency reactivator *in vitro*, assessed in two primary T cells latency models. One major concern of HIV-1 reactivation during “shock and kill” approaches is still the infection of bystander lymphocytes despite of ART^11^. Although used as a reactivator, MVC would also block the entry of the R5-tropic newly reactivated viruses and consequently the probability of exposure and the viral vulnerability to the “kill” strategy would be increased. In this regard, reactivated viruses are expected to be mainly R5-tropic variants as far as most founder viruses establishing the latent reservoir during early infection use CCR5 as a co-receptor^73^. An additional encouraging feature of MVC as a viral reactivator is that drug safety in humans is well-known due to current therapeutic use. However, our findings present major limitations and raise some questions that should be answered in future studies. *Ex vivo* assays in rCD4+ T lymphocytes isolated from infected patients would confirm that MVC is able to mobilize latent reservoirs *in vivo* and should be performed prior to clinical trials. Combination of MVC with other LRAs, as JQ1 or HDACi among others, may be of importance to find synergistic actions with potential therapeutic approaches.

## Materials and Methods

### Cells

Peripheral blood mononuclear cells (PBMCs) were isolated from healthy donors by Ficoll-Hypaque gradient (GE Healthcare). Human rCD4+ T lymphocytes were isolated using CD4+ T Cell Isolation Kit (Miltenyi Biotec) and subsequent depletion of CD25+ and HLA-DR + cells using respectively CD25 MicroBeads II and anti-HLA-DR MicroBeads (Miltenyi Biotec). CD4+ Central Memory Isolation kit was used to isolate TCM cells.

### Reagents and vectors

CCL19 and IL-7 (R&D Systems) were used at 29 nM and 1 nM, respectively. IL-2 was used at 10 IU/mL (Sigma-Aldrich). PMA and PHA (Sigma-Aldrich) was used at 12.5 ng/ml and 0.5 ug/mL, respectively. Different concentrations of MVC (Sigma-Aldrich) ranging from 0.04 μM to 80 μM were used, but 5 μM of MVC was used in most experiments. Bryostatin-1 (Sigma-Aldrich) was used at 1uM, 100 nM, 10 nM or 1 nM. pLTR-LUC vector was already described^74^. CD195 Mouse anti-Human conjugated to PE (clone 2D7) for staining CCD5 receptor was purchased from BD Biosciences.

### Cell proliferation

The cell ability to proliferate was measured in rCD4+ T cells treated with MVC (5 μM) alone or combination with Bryostatin-1 (10 nM or 100 nM) for 18 h by quantifying active DNA synthesis. Non-treated or PHA-treated cells were used as a negative or a positive control, respectively. The commercial assay Click-iT® EdU for Flow Cytometry was used (Invitrogen). EdU was added to the culture medium at 10 μM for 2 hours. Cells were then fixed with and permeabilized prior to stain with the detection reagent coupled to AlexaFluor-488 dye. The percentage of S-phase cells was quantified by flow cytometry. Data acquisition was performed in a FACScalibur Flow Cytometer (BD Biosciences) using FL-1 channel. Data analysis was done using CellQuest software. Fluorescence from non-treated cells that were not incubated with EdU was subtracted from each experimental condition.

### Cell viability

Cell viability was measured in cells treated with MVC and/or with Bryostatin-1 using CellTiter-Glo Luminescent Cell Viability Assay (Promega). Brifely, 2 × 10^5^ cells were incubated for 10 min at room temperature in CellTiter-Glo® Reagent, which contained lysis buffer and thermostable luciferase. The luminescent signal was analyzed in an Orion Microplate Luminometer with Simplicity software (Berthold Detection Systems).

### Cellular transfections

rCD4+ T cells were transiently transfected with pLTR-LUC using Gene Pulser electroporator (BioRad). In brief, 20 × 10 rCD4+ T cells were collected in 350 uL of RPMI 1640 medium without supplements and mixed with 1 ug/106 cells of plasmid pLTR-LUC. Cells were transfected in a cuvette with a 4-mm electrode gap (EquiBio) at 320 V, 1500 microfarads and maximum resistance. Four hours post-transfection, cells were challenged with different concentrations of MVC. After transfection, cells were incubated in supplemental RPMI at 37 °C for 4 h and the stimulated with different concentrations of MVC for the following 18 h. Luciferase activity was assayed using Luciferase Assay System (Promega), according to manufacturer’s instructions. Briefly, cells were lysed and incubated with equal volume of substrate. Relative Luciferase Units (RLUs) were measured in supernatants with a Sirius luminometer (Berthold Detection Systems). RLUs were normalized by measuring the total protein.

### Latent HIV-1 infections

Latent HIV-1 infection of purified primary rCD4+ or TCM cells was performed as previously described with minor modifications^18–21^. Cells were cultured for 5 days in the presence of CCL19 (29 nM) or IL-7 (1 nM) or left inactivated. HIV-1 infection was previously described^75^. Infectious supernatants were obtained from calcium phosphate transfection of 293T cells with HIV-1 plasmids and were treated with DNAse (Qiagen) before infection. Cells were infected with 10 pg of p24 per million cells of X4-tropic NL4–3 or with R5-tropic BaL infectious supernatants for 2 hours with gentle rotation at room temperature. Cells were then centrifuged at 600 g for 30 min at 25 °C. After extensive washing with 1× PBS, infected cells were cultured in RPMI supplemented with IL-2 (10 IU/mL) for an additional 5 days. CCL19 or IL-7 was also added as indicated. Integrated HIV-1 was quantified as previously described^5, 76^. Briefly, strong stop DNA quantified using primer pairs specific for R and U5 regions of the HIV LTR. Eppisomal forms of 2-LTR and integrated HIV-1 proviral DNA were quantified by PCR using a LightCycler 480 Instrument II (Roche). A standard curve of integrated DNA from 8E5 cell line was prepared, and ccr5 gene was used as housekeeping. For reactivation assays, stimuli were added during the last 18 h. Supernatants were collected, and HIV-1 p24 antigen was measured in the culture supernatants by using an enzyme-like immunoassay (InnotestTM HIV Ag mAb; Innogenetics).

### Analysis of CCR5 expression

CD4 cells isolated from healthy donors were treated with IL-7 for 10 days or left untreated as referred control. Cells were then stained with a monoclonal antibody against CCR5 conjugated to PE or with the matching isotype control. Data acquisition was performed in a FACScalibur Flow Cytometer using FL-2 channel. Data analysis was done using CellQuest software.

### Ethical statement

Fresh human whole blood samples used in this study were obtained from healthy anonymous volunteers at the Transfusion Center of the Community of Madrid (Spain). Proper informed consent was obtained from each subject in accordance with the Spanish legislation on blood donor regulations. Confidentiality and privacy was assured. The clinical research ethics committee of the Instituto Ramón y Cajal de Investigación Sanitaria (IRYCIS) approved the use of human blood samples for this study. All procedures used were in accordance with the ethical standards on human experimentation established by IRYCIS ethics committee, which are based on the Helsinki Declaration.

### Statistical analysis

Statistical analysis was calculated with Graph Pad Prism 5.0 (San Diego, CA) using non-parametric with Kruskal-Wallis Test and Dunn’s Multiple Comparison Test. Comparisons between more than two groups were performed with two-way analysis of variance (ANOVA) with Bonferroni post-test analysis.
